# Hybrid algorithm for the detection of turbulent flame fronts

**DOI:** 10.1007/s00348-023-03651-6

**Published:** 2023-05-19

**Authors:** Oussama Chaib, Yutao Zheng, Simone Hochgreb, Isaac Boxx

**Affiliations:** 1 Department of Engineering, Cambridge University, Trumpington Street, Cambridge, CB2 1PZ UK; 2 Institut für Verbrennungstechnik, Deutsches Zentrum für Luft- und Raumfahrt (DLR), 70569 Stuttgart, Germany; 3 Chair of Optical Diagnostics for Energy, Process and Chemical Engineering, RWTH Aachen University, Aachen, Germany

## Abstract

**Abstract:**

This paper presents a hybrid and unsupervised approach to flame front detection for low signal-to-noise planar laser-induced fluorescence (PLIF) images. The algorithm combines segmentation and edge detection techniques to achieve low-cost and accurate flame front detection in the presence of noise and variability in the flame structure. The method first uses an adaptive contrast enhancement scheme to improve the quality of the image prior to segmentation. The general shape of the flame front is then highlighted using segmentation, while the edge detection method is used to refine the results and highlight the flame front more accurately. The performance of the algorithm is tested on a dataset of high-speed PLIF images and is shown to achieve high accuracy in finely wrinkled turbulent hydrogen-enriched flames with order of magnitude improvements in computation speed. This new algorithm has potential applications in the experimental study of turbulent flames subject to intense wrinkling and low signal-to-noise ratios.

**Graphic abstract:**

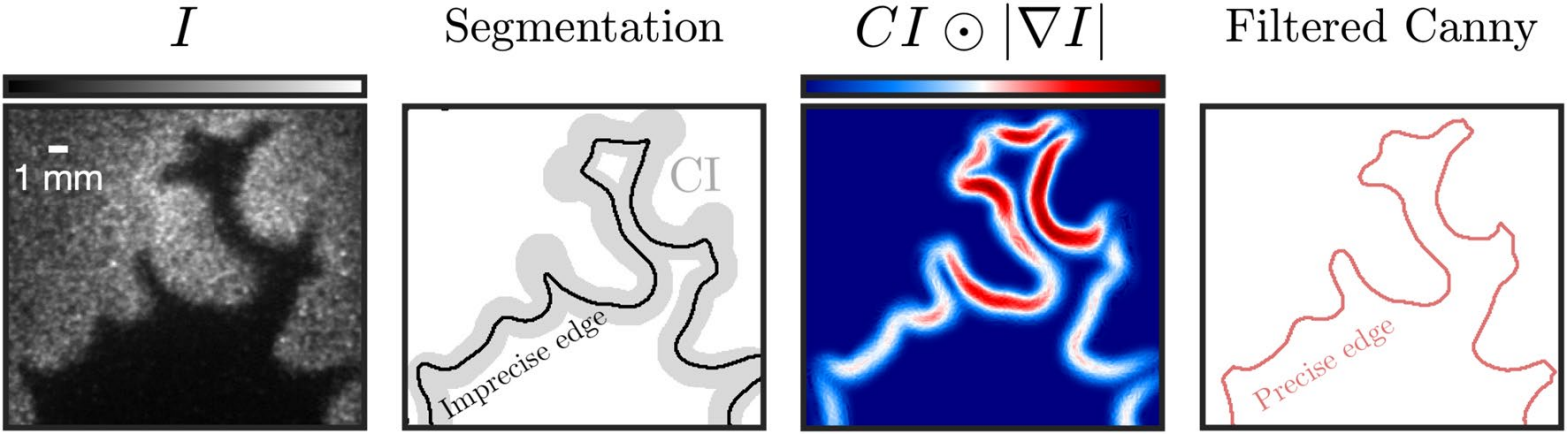

**Supplementary Information:**

The online version contains supplementary material available at 10.1007/s00348-023-03651-6.

## Introduction

Identifying the instantaneous position of the flame front and its representative geometric features is one of the most common tasks in the post-processing of optical images of turbulent flames. Indeed, accurate flame front detection gives access to an array of quantities such as curvature and flame surface density which are essential to relating turbulence and reaction rates. A common definition of the flame front is the location of peak heat release (Sweeney and Hochgreb 2009). Direct, spatially resolved measurements of heat release rate are hardly feasible; instead, alternatives such as planar Rayleigh scattering can be used to measure two-dimensional temperature fields. This method is, however, known to yield low signal-to-noise ratio (SNR) images and presents a number of experimental difficulties when simultaneous particle-based velocity measurements are desired (Sweeney and Hochgreb 2009; Pfadler et al. 2007). Planar laser-induced fluorescence of the hydroxyl radical (OH-PLIF) is therefore a popular alternative, wherein the flame front is determined by locating the steepest gradients of OH intensity (Sweeney and Hochgreb 2009). This is generally considered a valid tracer of the flame front as local maxima of OH gradients tend to be reasonably close to the location of peak heat release (Sweeney and Hochgreb 2009; Pfadler et al. 2007). A primary concern in flame front detection tasks, however, especially at high repetition rates, is the SNR which can be quite low due to variations in laser beam energy. This results in a non-homogeneous intensity profile across the same image and intensity fluctuations on a shot-to-shot basis. To address this issue, common practice is either to use pixel binning or Gaussian filtering of the images, which results in an inevitable loss of spatial resolution (Boxx et al. 2015). Other difficulties stem from the convoluted nature of turbulent flames, often featuring a number of reactant and product pockets in the corrugated flamelet regime (Tyagi et al. 2019, 2020). Additional challenges arise when thermodiffusively unstable (i.e., lean hydrogen/air) flames are investigated, which feature a wide range of cellular structures and thin elongated fingers (Berger et al. 2019, 2022; Day et al. 2009; Bell et al. 2007; Howarth and Aspden 2022). In these situations, common flame front detection algorithms may reach their limits, which highlights the need for more robust and systematic approaches. As a general principle, flame front detection algorithms should meet a set of essential criteria. First, the algorithm should accurately identify the location of the flame front (associated with peak gradients) and its key geometric features such as curvature. Second, the algorithm should require as little supervision as possible, meaning it is capable of adapting its parameters autonomously based on the quality and SNR of the image it is given. Third, the algorithm should be computationally undemanding and therefore practical for processing larger image sets from high-speed diagnostics.

In this work, we examine and compare the two common routes to flame front detection, namely *segmentation* and *edge detection*, in processing low-SNR OH-PLIF images of turbulent hydrogen-enriched flames. A pre-processing scheme based on adaptive contrast adjustment is proposed to improve the accuracy of segmentation. We then propose a hybrid edge detection method, the *Filtered Canny algorithm*, combining both segmentation and edge detection for accurate, unsupervised, and low-cost flame front identification. The algorithm is evaluated against a high-performance, computationally intensive alternative, the Augmented Canny algorithm (Sweeney and Hochgreb 2009), and is shown to produce results of comparable accuracy with a considerable reduction in computational time. The proposed method showcases excellent accuracy in hydrogen-rich cases and is able to accurately trace the shape of the flame front and detect all isolated flame pockets.

## Experimental methodology

**Table 1:** Experimental conditions investigated in this study

Case	$\chi _{H_2}$ (%)	$\varPhi$ (−)	$s_L$ (m s$^{-1}$)	$\delta _L$ (mm)	$U_{0}$ (m s$^{-1}$)	$u'$ (m s$^{-1}$)	$l_t$ (mm)	$Re_T$ (−)	Ka (−)
1	0	1.00	0.38	0.44	2.13	0.46	3.20	93.81	1.61
2	40	0.80	0.38	0.44	2.18	0.48	3.30	100.96	1.69
3	70	0.65	0.35	0.47	2.12	0.45	3.20	92.78	1.81

The laminar flame speed and (thermal) flame thickness, denoted $s_L$ and $\delta _L$, respectively, were obtained from 1D Cantera simulations of freely propagating unstretched laminar flames using the GRI-Mech 3.0 mechanism (Goodwin et al. 2022; Smith et al. 1999). The bulk flow velocity $U_0$ was estimated based on the total mass flow rate of reactants $\dot{m}$. Velocity fluctuations were estimated using the root-mean-square radial and axial components of velocity $u' = \sqrt{{u'^2_r}+{u'^2_z}}$, measured by particle-image velocimetry (PIV) (Pareja et al. 2022). The integral lengthscale was obtained from scaling estimates $l_t \approx \frac{u'}{U_0}d$. The turbulent Reynolds $Re_T$ and Karlovitz *Ka* numbers were estimated assuming a mixture density equal to that of air: $\nu = 15.69\times 10^{-6}$ m$^2$ s$^{-1}$

Measurements were conducted in flames generated with the DLR Bunsen Burner (Pareja et al. 2022). This burner has an internal geometry similar to that used by Coppola and Gomez (2009). It consists of a cylindrical plenum that terminates at a high blockage ratio turbulence generator plate, which is followed by a straight conical contraction. The nozzle has a contraction angle of 15$^{\circ }$, followed by a straight section of 10 mm. It has an outlet diameter of $d = 15$ mm. The turbulent generator plate located at the base of the conical contraction has four circular holes (4.8 mm in diameter) evenly spaced around a ring of 36 mm in diameter, which yields a blockage ratio of 96%.

Experiments were conducted at atmospheric conditions (1 atm, 300 K) and are presented in Table 1. An initial methane-air flame (Case 1) was stabilized on the DLR Bunsen Burner and then enriched with a variable amount of hydrogen (Cases 2 and 3). Hydrogen enrichment (denoted $\chi _{H_2}$) is defined here as the volume fraction of hydrogen in the cold fuel mixture. The burner was operated at low velocity and turbulence characteristics were kept constant across all the conditions investigated. The unstretched laminar flame speed was kept constant by tuning down equivalence ratios ($\varPhi$) as hydrogen was added to the reactant flow.

Flames in this burner were imaged using a high-speed (10 kHz acquisition rate) OH-PLIF imaging system. This system has been described extensively in previous papers (Kushwaha et al. 2021). For completeness, a brief description is provided here. The OH-PLIF system is based on a frequency-doubled dye laser, pumped by a highspeed, pulsed Nd:YAG laser (Edgewave IS400-2-L, 150 W at 532 nm and 10 kHz) and an intensified high-speed CMOS camera system. The dye laser system (Sirah Credo) produces approximately 5.3 to 5.5 W at 283 nm and 10 kHz repetition rate (i.e., 0.53$-$0.55 mJ/pulse). The dye laser was tuned to excite the $Q_1(6)$ line of the $A^2\varSigma ^+-X^2 \varPi$ ($\nu ^\prime$= 1, $\nu ^{\prime \prime }$= 0) band. The PLIF excitation beam is formed into a sheet approximately 50 mm (high) $\times$ 0.2 mm (thick) using three fused-silica, cylindrical lenses (all anti-reflective coated to maximize transmission). OH-PLIF fluorescence signal was imaged using a high-speed CMOS camera (LaVision HSS6) and external two-stage intensifier (LaVision HS-IRO). The OH-PLIF camera was equipped with a UV-capable objective lens (Cerco, f = 100 mm, f/2.8) and a bandpass filter (300–325 nm). The projected pixel resolution of the camera was 0.07 mm/px, with an array size of $768\times 768$ px$^2$. A total of 19,400 8-bit grayscale images were collected for each experimental condition. Mean values of the SNR ranged between 1.9 and 2.5 in this study, with recorded standard deviations between 0.15 and 0.2. These were estimated from raw images using a similar definition to the one in Sweeney and Hochgreb (2009) with additional details provided in “Appendix.”

## Flame front detection

**Fig. 1:**
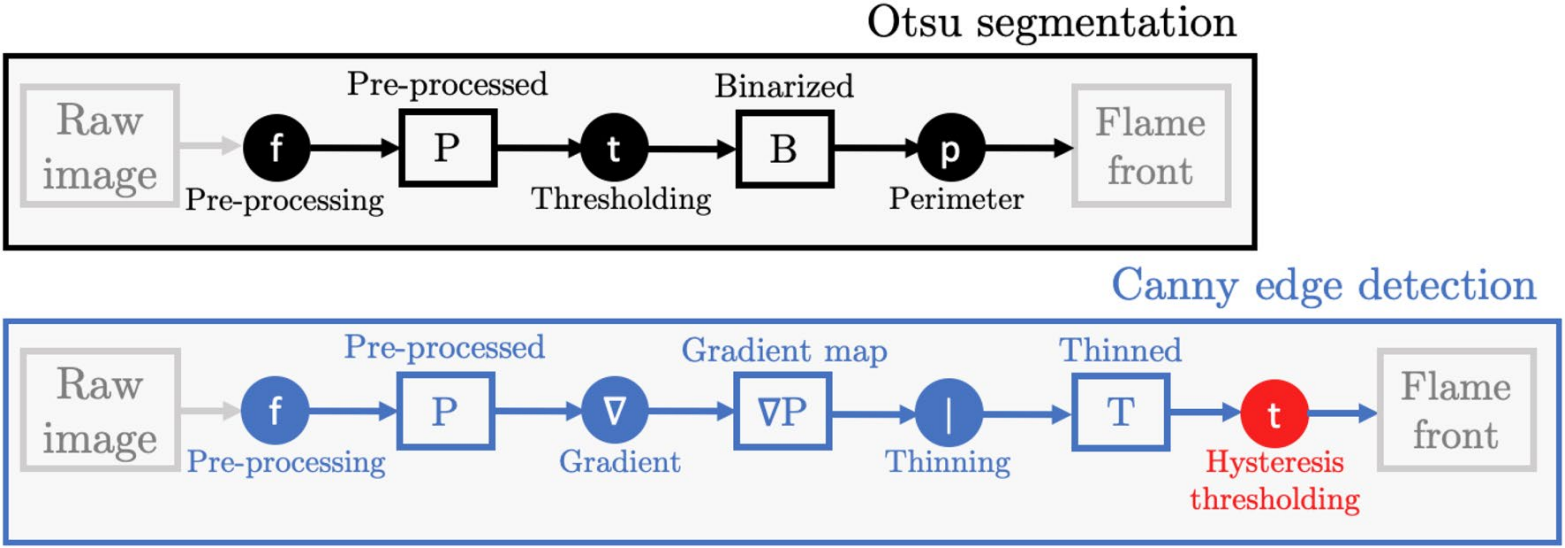
Flowchart of flame front detection algorithms. Stages requiring supervision are shown in red

The vast majority of flame front detection algorithms in experimental combustion literature fall under two categories: segmentation or edge detection. In this section, we briefly discuss the background of the two most popular state-of-the-art algorithms: Otsu segmentation and Canny edge detection (Fig. 1).

### Segmentation

**Fig. 2:**
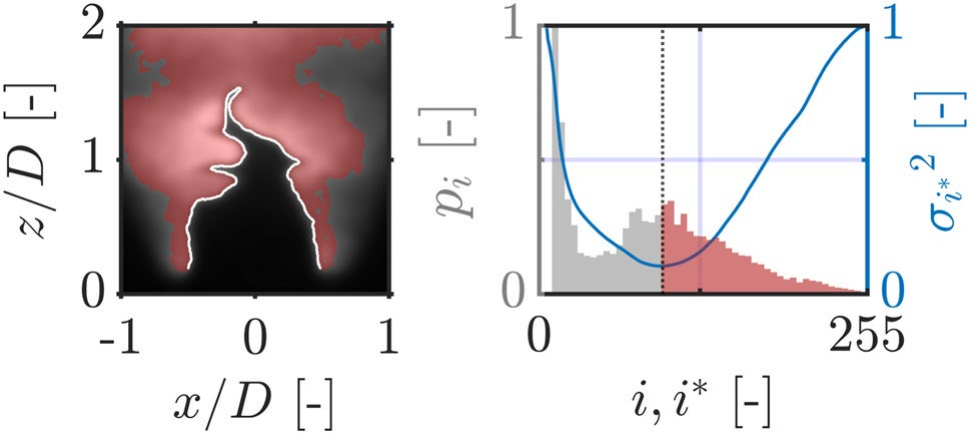
Illustration of a segmentation operation applied to an OH-PLIF image of a turbulent premixed hydrogen-enriched methane-air flame (left) and the associated normalized image histogram (right). Each bar in the image histogram represents the frequency of a given intensity *i* in the 8-bit grayscale PLIF image, normalized to the [0, 1] range, $p_i$. The dark vertical dotted line is the optimal (Otsu) threshold corresponding to the global minimum of the intra-class variance $\sigma _{i^*}^2$ traced in blue color. This threshold partitions the PLIF image into two distinct regions highlighted in red (burnt) and gray (unburnt), respectively, with the flame front (white) separating both

Segmentation is particularly attractive for its simplicity and low computational cost. It relies on binarizing the image into two distinct regions corresponding to a foreground (burnt gases) and a background (unburnt gases). Given an arbitrary intensity threshold $i^*$, pixels are classified as either background ($i\le i^*$) or foreground ($i>i^*$) pixels based on their intensities *i*. The boundary or perimeter of the binarized image is then labeled as the flame front (Fig. 1). Many past investigators have made use of segmentation by setting an intensity threshold $i^*$ manually (Kobayashi et al. 2005; Zhang et al. 2014; Mohammadnejad et al. 2021; Tachibana et al. 2004; Gulder and Smallwood 2007; Lawn and Schefer 2006; Wabel et al. 2017) or statistically based on the image histogram (Tyagi et al. 2020; Halter et al. 2009; Malbois et al. 2019; Fan et al. 2022). The latter approach is referred to as Otsu segmentation and can be used to estimate an optimal binarization threshold dynamically and fully unsupervised (Otsu 1979). The method originally proposed in the pioneering work of Otsu (1979) assumes the image histogram (Fig. 2) follows a bimodal distribution and tries to identify the optimal value of $i^*$ which ensures both are “maximally separated” (Burger and Burge 2016). Separability is assessed using a discriminant criterion known as the intra-class (or within-class) variance (Otsu 1979): for a given intensity threshold $i^*$, the intra-class variance $\sigma _{i^*}^2$ is a weighted sum of the variances of background and foreground pixel intensities:

1 $$\begin{aligned} \sigma _{i^*}^2 = \sum _{c=0}^1 \omega _c \sigma _c^2 = \omega _0 \sigma _0^2 + \omega _1 \sigma _1^2 \end{aligned}$$

where $\omega _c$ and $\sigma _c^2$ are the probability and variance of class *c*, respectively, $c = 0$ being background pixels ($i\le i^*$) and $c=1$ foreground pixels ($i>i^*$) (Otsu 1979). The probability $\omega _c$ refers to the cumulative distribution function of class *c*, or simply the proportion of pixels in *c* out of the total number of pixels which make up the image (with $\sum _{c=0}^1 \omega _c = \omega _0 + \omega _1 = 1$). The algorithm identifies the optimal threshold as the value of $i^*$ which minimizes the intra-class variance $\sigma _{i^*}^2$ and hence yields two narrow and well-separated distributions with minimal variances. This is illustrated in Fig. 2 for an OH-PLIF image of a methane-air flame with moderate hydrogen enrichment (Case 2).

Despite its low cost and lack of supervision, Otsu segmentation tends to be less accurate as the flame fronts obtained are usually shifted from the location of peak gradients, as has been observed in Sweeney and Hochgreb (2009). As will be seen later, these shifts can be significant and are non-trivial as they can result in inaccurate estimation of flame surface area, particularly when flames are subject to intense wrinkling. These inaccuracies are inherent to the algorithm as the obtained flame front is more sensitive to the shape of the global image histogram than the sudden jumps in OH intensity near the reaction zone. This raises questions on the accuracy of segmentation, knowing histograms can be easily affected by nonhomogeneities caused by the laser beam energy or the variable concentration of OH in the post-combustion region.

### Edge detection

Edge detection is a common alternative to segmentation in flame front detection tasks. The aim is to locate the steepest gradients of OH intensity in PLIF images, which makes it a more accurate tracer of the position of the flame front. It is also a more attractive approach when the PLIF signal varies across the image, as gradients tend to be more reliable and resilient to noise than raw intensity values. Basic edge detection relies on computing a two-dimensional gradient map of the image, then using gradient magnitudes and their direction to trace a flame front and its relevant geometric features (Burger and Burge 2016). Many methods exist (Sobel, Prewitt, LoG, etc.) and have been used to locate the flame front from OH-PLIF imaging data (Burger and Burge 2016; Bayley et al. 2012; Boxx et al. 2015; Stöhr et al. 2012; Verbeek et al. 2013). Among these methods, the Canny edge operator, initially developed by John Canny in 1986 (Canny 1986), continues to be a state-of-the-art go-to edge detection algorithm and remains the most popular choice in experimental combustion literature (Sweeney and Hochgreb 2009; Hartung et al. 2009; Sweeney et al. 2011; Wabel et al. 2017; Zheng et al. 2022). In a comparative study (Reisenhofer et al. 2016), it was also found to be relatively resilient to noise, yielding comparable results to more sophisticated algorithms based on shearlets, when applied to artificially distorted OH-PLIF images.

**Fig. 3:**
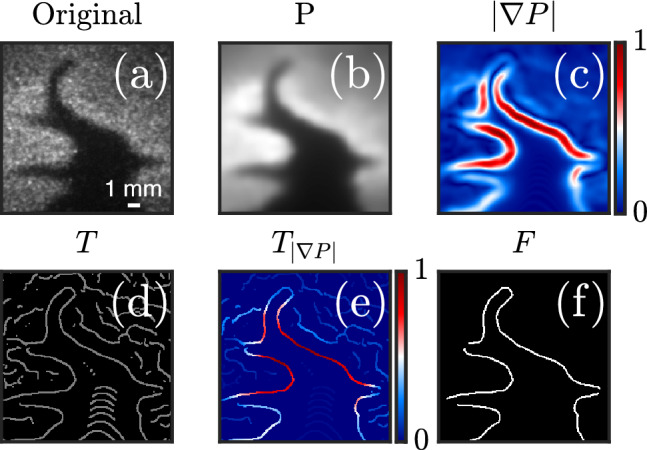
Illustration of the Canny edge detection process: **a** Raw OH-PLIF image (zoomed section from the snapshot in Fig. 2), **b** Pre-processed image ($\sigma = 2$), **c** Two-dimensional gradient map of the filtered image, colors correspond to gradient magnitudes normalized to the [0, 1] range, **d** Thinned gradient map, **e** Gradient magnitudes of the thinned gradient map, **f** Final binary flame front after hysteresis thresholding. Optimal parameters ($t_{\textrm{low}} = 0.19$, $t_{\textrm{high}} = 0.35$) were determined by trial and error

The algorithm (Fig. 1) can be broken down to four steps illustrated in Fig. 3:

1. *Pre-processing:* The image *I*(*x*, *z*) is smoothed by convolution (denoted $\circledast$) with a Gaussian kernel *G* of width $\sigma$ defined by the user.
  2 $$\begin{aligned} {\left\{ \begin{array}{ll} P = G_\sigma \circledast I\\ G_\sigma (x,z) = \frac{1}{2\pi \sigma ^2} e^{-\frac{x^2+z^2}{2\sigma ^2}} \end{array}\right. } \end{aligned}$$
  where *x* and *z* correspond to the radial and axial coordinates, respectively.
2. *Gradient computation:* The two-dimensional gradient of the smoothed image $|\nabla P|$ is computed by convolution with a gradient kernel, then normalized to the [0, 1] range.
3. *Thinning / Non-maxima suppression (NMS):* The gradient map is thinned to produce an image *T* consisting of edges of single pixel width. This is typically done by placing a $3\times 3$ px$^2$ box at each pixel and comparing its gradient magnitude $|\nabla P|$ to that of its adjacent pixels in the direction of the gradient $\theta$.
  3 $$\begin{aligned} \theta = \arctan \frac{\nabla _x P}{\nabla _z P} \end{aligned}$$
  The local gradient magnitude is kept only if it is higher than its neighbors in the direction given by $\theta$. This ensures only local maxima are preserved.
4. *Hysteresis thresholding: * This step aims at eliminating low gradient edges due to noise and non-uniformities. Two hysteresis (gradient) thresholds, $t_{\textrm{low}}$ and $t_{\textrm{high}}$, are defined by the user. All pixels in *T* with gradient magnitudes $|\nabla P|$ higher than $t_{\textrm{high}}$ are highlighted as part of the flame front $F_a$. Then, pixels whose gradient magnitudes are situated between $t_{\textrm{low}}$ and $t_{\textrm{high}}$ are classified as flame edge pixels so long that they are adjacent to previously defined flame edge points in $F_a$. Hence, a final flame front *F* is obtained.
  4 $$\begin{aligned} {\left\{ \begin{array}{ll} F_{a} = T_{|\nabla P| \ge t_{\textrm{high}}} \\ F_{b} = T_{|\nabla P| \in [t_{\textrm{low}}\,;\,t_{\textrm{high}})} \\ F = F_a\, \cup \, \left[ F_b \cap (d_{(F_{b},F_{a})} \le \epsilon )\right] \end{array}\right. } \end{aligned}$$
  where $d_{(F_{b},F_{a})}$ is the distance between each point in $F_b$ and its closest point in $F_a$, $\epsilon = \sqrt{2}$ px is the minimum distance for adjacency (i.e: 8-connectivity). This process tends to be more effective than setting one rigid gradient threshold, as OH gradient magnitudes can vary significantly across the flame front.

**Fig. 4:**
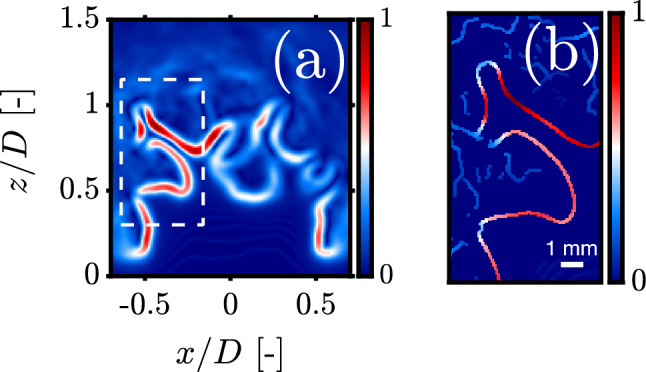
**a** Two-dimensional gradient map for an OH-PLIF image at 70% hydrogen enrichment normalized to the [0, 1] range, **b** Thinned gradient map of a high SNR region (dashed white box in **a**) after NMS

At this stage, it is important to mention that the Canny algorithm, unlike Otsu segmentation, is supervised and requires that three operating parameters ($\sigma$, $t_{\textrm{low}}$, and $t_{\textrm{high}}$) be specified by the user. Provided that a robust filtering scheme is implemented in the pre-processing stage preceding edge detection, $\sigma$ can be set to a low and constant value (i.e., $\sigma =2$ in this work) with negligible effect on the final result. The same cannot be said about the hysteresis thresholds, $t_{\textrm{low}}$, and $t_{\textrm{high}}$. In fact, the selection of optimal thresholds constitutes a real challenge as they are ultimately the deciding factor in the success (or failure) of the flame front detection. Approaches based on a priori selection of a single combination of $t_{\textrm{low}}$ and $t_{\textrm{high}}$ are known to perform poorly when the SNR is low and variable (Sweeney et al. 2011). Notice, for example, the large variance in gradient magnitudes across the flame front due to noise in Figs. 3c, e and 4a. Hence, accuracy and robustness entail that both parameters be selected dynamically on an image-by-image basis, and autonomously such that large datasets can be processed within reasonable computation times. However, and to the authors’ best knowledge, there is currently no state-of-the-art, fast, and unsupervised method of determining the optimal gradient thresholds. Some authors have documented the use of the Otsu threshold to predict both parameters (Azam et al. 2020; Setiawan et al. 2017). This usually involves setting $t_{\textrm{high}}$ to the Otsu threshold and $t_{\textrm{low}}$ to a fraction of it, usually one-half. This is a clever way of computing hysteresis thresholds unsupervised, but the flame fronts obtained using this approach were deemed unsatisfactory. A more sophisticated method, the Augmented Canny algorithm, was demonstrated by Sweeney and Hochgreb (2009) and tested on OH-PLIF images of variable SNR. The algorithm treats parameter selection as a search-based optimization problem against a statistically derived ground-truth. This was found to produce excellent results but at high computational cost, as highlighted by the original authors. This makes it unattractive for processing larger data sets which is key in investigations involving data from high-speed diagnostics.

Hence, in spite of its excellent accuracy, edge detection requires reasonable supervision to ensure optimal parameters are chosen. Methods based on autonomous parameter selection, like the Augmented Canny algorithm, can help address this downside but at the expense of computational time, which is undesirable when processing large batches of experimental data.

### Hybrid segmentation and edge detection

At this stage, a few interesting observations can be made. We have on the one hand a quick, unsupervised, but inaccurate algorithm (Otsu segmentation) and on the other hand an accurate but supervised algorithm (Canny edge detection) which can only run unsupervised at the cost of precious computation time. In light of this, it is clear that a hybrid method combining both approaches would be of interest. The goal would be to use the approximate location of the flame front obtained using segmentation to guide the edge detection algorithm and eliminate the need for supervision. This involves finding a way around the problematic hysteresis thresholding stage which is the major point of supervision in the Canny algorithm. This idea is further explored in Sect. 4.3 and forms the basis of the hybrid *Filtered* Canny algorithm proposed in this work.

A second motivation for a segmentation and edge detection hybrid stems from the thermodiffusive nature of lean hydrogen-air flames which causes the concentration of the OH radical to vary with the local curvature of the flame front. As can be seen in Fig. 4b, gradients of the OH-PLIF signal can reach global minima when the flame front is concave toward reactants which is likely due to a lower OH concentration in the reaction layer as observed in direct numerical simulations (Bell et al. 2007). Gradients at the tip of these flame fingers are of comparable magnitude to the spurious edges. This adds an extra layer of difficulty to hysteresis threshold selection, knowing the noisy nature of images poses challenges of its own (Fig. 4a). In this case specifically, no single combination of parameters $t_{\textrm{low}}$ and $t_{\textrm{high}}$ was able capture the accurate shape of the flame front. Hence, there is a real need for alternatives to hysteresis gradient thresholding when dealing with thermodiffusively unstable mixtures.

### The importance of pre-processing

Prior to flame front detection, it is common practice to pre-process images using a suitable combination of filters and contrast enhancement techniques. The goal is to improve the quality of the raw images and reduce some of the experimental noise to render any further processing possible. Filtering the image also helps produce smoother flame fronts unpolluted by high-frequency noise and undesirable zigzag effects. Here, we briefly highlight the importance of ensuring compatibility between flame front detection and pre-processing techniques, which is often overlooked in combustion literature.

**Fig. 5:**
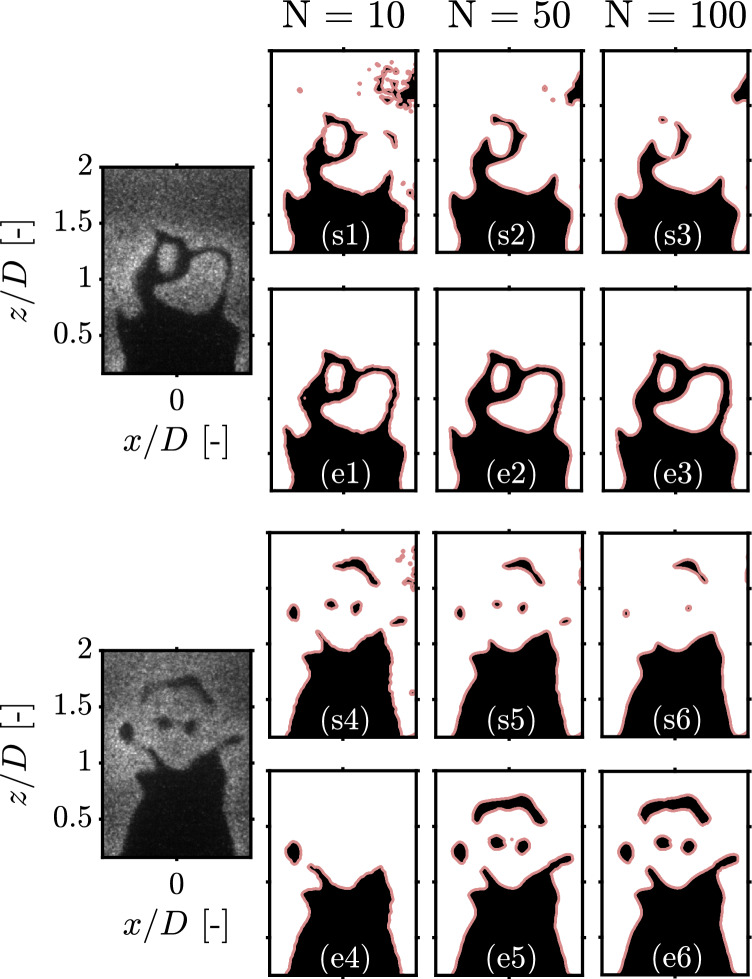
Effect of NLD filtering on the flame fronts obtained using Otsu segmentation (s1-6) and Canny edge detection (e1-6) for two different OH-PLIF snapshots. The number of filtering iterations *N* is varied from low ($N=10$) to high ($N=100$) filtering. The flame fronts obtained using segmentation and edge detection are traced in red with the associated burnt and unburnt gas regions shown in white and black color, respectively

A wide variety of filters have been used to process PLIF images in the past, ranging from simple linear filters based on averaging or low-pass filtering (moving mean, Gaussian, Wiener) to more sophisticated non-linear, edge-preserving, filters (median, bilateral, nonlinear diffusion, wavelet...) (Boxx et al. 2015; Tyagi et al. 2020; Mohammadnejad et al. 2021; Lawn and Schefer 2006; Bayley et al. 2012; Skiba et al. 2022; McManus and Sutton 2020; Barlow 2007). The latter tend to be preferred over their linear counterparts which can significantly degrade the image resolution and smear its characteristic features such as gradients and curvature (Malm et al. 2000). Edge-preserving filters are attractive as their smoothing behavior varies based on the local structure of the image. In other words, smoothing is maximum in uniform regions and tuned down as high gradient regions of the image are approached (Burger and Burge 2016). This allows for considerable noise reduction in the burnt and unburnt gas regions while preserving the location and magnitude of gradients in the vicinity of the reaction zone. It is important to state that edge-preserving filters, which are gradient-sensitive by definition, show good compatibility with edge detectors but not with segmentation techniques. This confusion stems from an abuse of language, wherein the term “edge detection” is used to refer to gradient and non-gradient methods interchangeably. This is illustrated in Fig. 5 for OH-PLIF snapshots filtered using non-linear diffusion (NLD), a state-of-the-art edge-preserving filter particularly popular in experimental combustion literature (Malm et al. 2000; Perona and Malik 1990). The chosen snapshots feature multi-scale product or reactant pockets which should be detected alongside the main flame front. The number of iterations of filtering *N* is varied to cover a reasonable range of low, moderate, and high filtering settings. The flame fronts obtained using segmentation (s1-6) are strikingly sensitive to filtering and indicate significantly different flame surface geometries depending on filtering intensity. In the first image, the thin corridors separating the isolated product pockets and the main flame front appear to shrink with increasing *N* which is likely due to the blurring effect of filtering. Notice how the flame surface in (s3) is almost unrecognizable from its original shape in the raw OH-PLIF image, as the two product pockets are absent and the algorithm detects two erroneous small reactant pockets downstream. Similar observations can be made about the second image, where the small reactant pockets shrink gradually with filtering until they disappear completely when *N* reaches 100. Conversely, flame fronts computed using Canny edge detection (e1-6) are remarkably resilient to filtering and become smoother as *N* is increased. They also seem to benefit from edge-preserving filtering in the second snapshot as the algorithm is able to detect the isolated reactant pockets with greater ease. This is an additional testament to the excellent compatibility between edge-preserving filtering and gradient-based flame front detectors. Hence, if edge-preserving filters are to be used jointly with segmentation as has been done in a handful past investigations (Sweeney and Hochgreb 2009; Tyagi et al. 2019, 2020; Malbois et al. 2019), caution must be exercised to avoid erroneous flame fronts.

Although less common than filtering, contrast enhancement methods have been used in a handful past experimental investigations, mostly looking at limit-phenomena (extinction and auto-ignition) where the SNR is typically very low (Qi et al. 2019; Manosh Kumar et al. 2019). The most basic contrast adjustment technique is histogram equalization (HE) which remaps the global image histogram to approximate a uniform distribution (Burger and Burge 2016). To avoid excessive noise amplification, adaptive histogram equalization (AHE) methods are used instead, where the image is divided into a number of boxes (or tiles) and HE is applied in each box individually using the local histogram. This results in a significant gain in SNR with relatively low noise amplification and ensures the global image histogram retains its characteristic bimodal distribution. A variant known as contrast-limited adaptive histogram equalization (CLAHE) (Zuiderveld et al. 1994; Pizer et al. 1986) is used in this work to improve the accuracy of segmentation and allow for accurate pocket detection. It was not used with edge detection as it was found to smear OH gradients across the flame front, which reiterates the importance of investigating compatibility between pre-processing and flame front detectors.

### Performance evaluation of flame front detection algorithms

Once the pre-processing is completed, the performance of flame front detection algorithms can be evaluated based on accuracy and computational time. The Augmented Canny algorithm (Sweeney and Hochgreb 2009) was used to generate a total of 1500 flame fronts (500 per experimental condition) which here are considered ground-truth (GT). These are the benchmarks for testing against the detected contours (DC) using the proposed algorithms. Accuracy is assessed in two different ways: localization errors and curvature differences.

Localization errors are assessed using the following metrics:

- *Euclidean distances*, *d*:
  5 $$\begin{aligned} d(a,b) = \sqrt{(x_b - x_a)^2+(z_b - z_a)^2} \end{aligned}$$
  where $a(x_a,z_a)$ is an arbitrary point in the detected contour *DC*, and $b(x_b,z_b)$ is the closest point to it in the ground-truth *GT*.
- *Precision* *and recall* using receiver operating characteristics (ROC) (Kraemer 1992) (Table 2). Here, precision represents the fraction of accurately identified edge points among the edge points reported, and recall denotes the fraction of accurately identified edge points out of the true edge points we expect to find. Both quantities are normalized between 0 and 1 by definition and maximum accuracy entails maximizing both. Precision and recall punish the algorithm in case of overestimation and underestimation of edge points, respectively.

While ROCs are strict metrics which penalize the algorithm if the DC and GT do not perfectly overlap, Euclidean distances can be used to measure the mean shift between both contours which offers additional leeway. Knowing the flame fronts obtained using segmentation do not necessarily correspond to the location of peak gradients which constitute the ground-truth obtained via the Augmented Canny algorithm, this ensures fair comparisons can be made. Moreover, as will be seen in Sect. 4.2, knowledge of Euclidean distance statistics is key to the implementation of the hybrid Filtered Canny algorithm proposed in this work.

**Table 2:** Receiver operating characteristic metrics used for accuracy evaluation

Symbol	Terminology	Definition
TP	True positive	$|\textrm{GT} \cap \textrm{DC}|$
TN	True negative	$|\overline{\textrm{GT}} \cap \overline{\textrm{DC}}|$
FP	False positive	$|\overline{\textrm{GT}} \cap {\textrm{DC}}|$
FN	False negative	$|{\textrm{GT}} \cap \overline{\textrm{DC}}|$
–	Precision	$\frac{\textrm{TP}}{\textrm{TP}+\textrm{FP}}$
–	Recall	$\frac{\textrm{TP}}{\textrm{TP}+\textrm{FN}}$

GT refers to the ground-truth (Augmented Canny algorithm), while DC refers to the detected contour using the relevant algorithm. Both GT and DC are binary sets. The modulus $|.\,|$ symbol refers to cardinality or the total number of pixels in the chosen set. The symbol $(\bar{.})$ refers to the complement of the chosen set

Two-dimensional curvature $\kappa$ is the second measure of accuracy considered in this study. It evaluates the algorithm’s ability to capture the overall shape or geometry of the flame front faithfully. Points along the flame front are indexed using a routine script and an arc length function *s*(*x*, *z*) is defined by measuring the distance across the flame front. The two-dimensional curvature can then be estimated using the formula:

6 $$\begin{aligned} \kappa = \frac{x'z''-x''z'}{(x'^2+z'^2)^{3/2}} \end{aligned}$$

where $x'=\frac{\textrm{d}x}{\textrm{d}s}$ and $z' = \frac{\textrm{d}z}{\textrm{d}s}\;$. Derivatives with respect to the curvilinear coordinate *s* are estimated at each point by fitting a second-order polynomial using the nearest 20 adjacent points (10 on either side) (Haq et al. 2002; Chrystie et al. 2008). The optimal order and number of points were determined by calibration against synthetic ground-truths (Chrystie et al. 2008). The usual sign convention is followed: Points are assigned positive curvature values if their center of curvature is located in the burnt gases and vice versa.

The final metric, computation time *t*, is estimated using MATLAB^®^’s stopwatch timer. The computation time will vary for each image based on the complexity of the flame front structure and the quality of the image. Therefore, we choose to record it for each snapshot independently so that reliable statistics can be computed.

## Hybrid flame front detection

The hybrid flame front detection method is presented in this section. The pre-processing scheme used to improve the performance of Otsu segmentation is first introduced. For brevity, this will be referred to as the *Enhanced* Otsu segmentation algorithm in the remainder of the paper. The accuracy of the improved scheme is then quantified using Euclidean distance statistics. We then demonstrate how this information can be used to develop a hybrid and unsupervised edge detection algorithm we refer to as the *Filtered Canny* algorithm.

### Enhanced Otsu segmentation

**Fig. 6:**
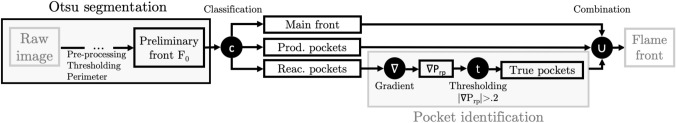
Flowchart of the enhanced Otsu algorithm

**Fig. 7:**
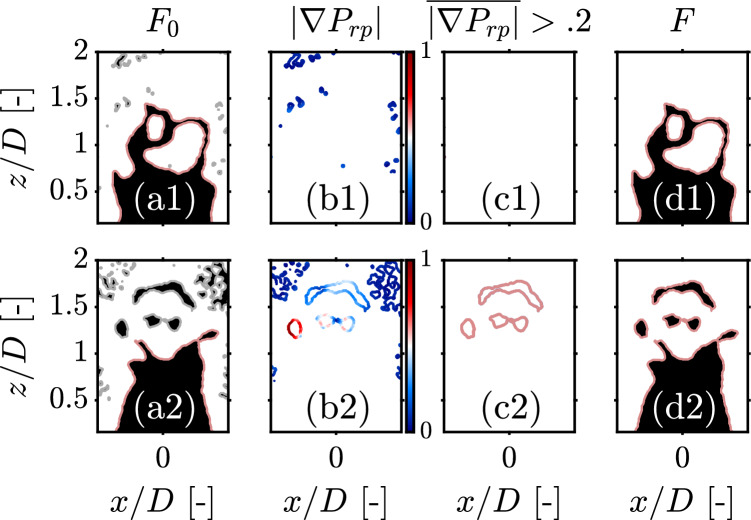
Pocket identification methodology for the two PLIF snapshots in Fig. 5: **a** Preliminary flame front $F_0$ after classification: the main front and product pockets are highlighted in red and reactant pockets are shown in gray color, **b** Two-dimensional gradient map of reactant pockets ${|\nabla P_{rp}|}$, **c** True reactant pockets of mean gradient $\overline{|\nabla P_{rp}|}>.2$, **d** Final flame front *F* after combining the main front, product pockets, and true reactant pockets

**Fig. 8:**
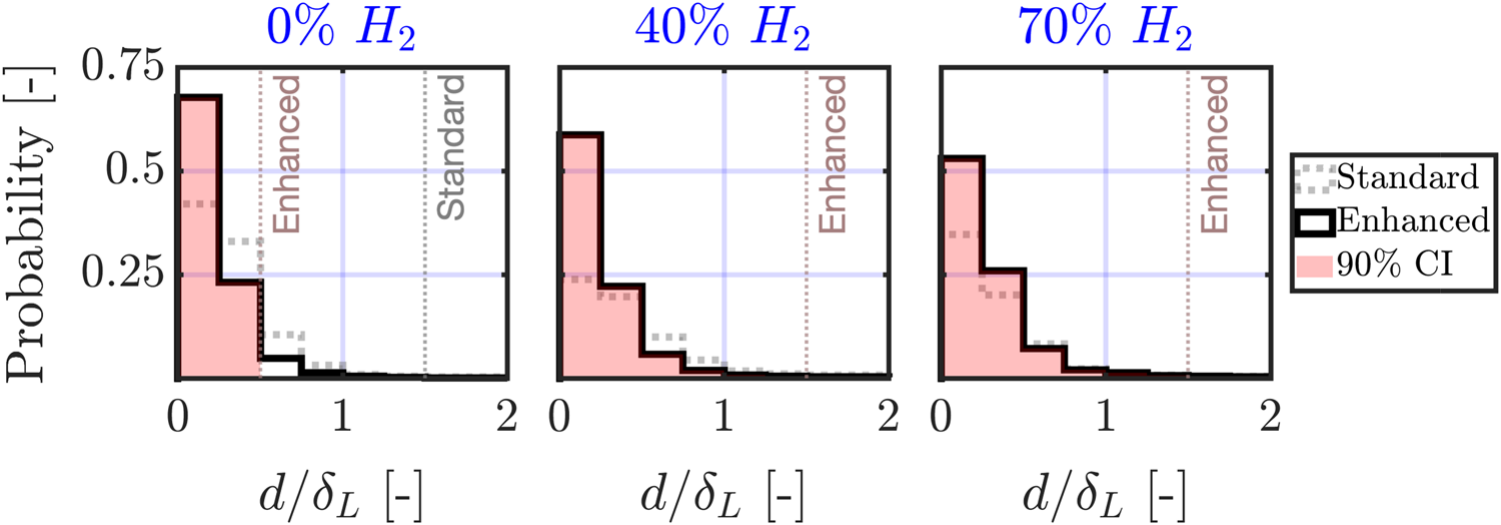
Histogram of Euclidean distances normalized by the laminar flame thickness $d/\delta _L$ for a variable hydrogen enrichment. Flame fronts obtained using the standard Otsu algorithm (dashed gray) are compared to those obtained using Enhanced Otsu segmentation (solid black). The 90% confidence interval (CI) relative to the Enhanced algorithm is overlayed in red. Dotted vertical lines represent the upper limits $d_{\textrm{CI}}$ of the $90\%$ confidence intervals. The upper limits of the standard algorithm do not appear in hydrogen histograms as they exceed the $d/\delta _L \in [0,2]$ range

The Enhanced Otsu segmentation process is illustrated in Fig. 6 and is similar to the original algorithm with two main differences. The first difference lies in the pre-processing stage where a contrast-enhancement scheme (CLAHE) is applied before filtering the image using NLD. The second difference lies in the two-stage nature of the algorithm (Fig. 6). As will be seen shortly, an additional “pocket identification” stage is required to ensure all reactant pockets are indeed true pockets and not noise artifacts.

In the pre-processing stage, MATLAB^®^’s implementation of CLAHE (adapthisteq) is used to adjust the contrast in the image. This is done by dividing the image into 64 equal tiles (8-by-8 grid) then applying histogram equalization to each one. About ${ClipLim = 1\%}$ of the total mass of each histogram is clipped and redistributed evenly across the full grayscale range to prevent over-saturation. The chosen settings are typical (Zuiderveld et al. 1994) and are kept constant for all the images processed in this work. Non-linear diffusion with quadratic settings and a low number of iterations (${N\le 20}$) is used to filter the image. The pre-processed image is then binarized using the Otsu threshold and an initial flame front is highlighted by selecting the perimeter.

In the second stage of the algorithm, flame edges are classified into three categories: main front, product pockets, and reactant pockets using simple image manipulation via MATLAB^®^ (more details on this process in “Appendix”). As can be seen in Fig. 7a, the algorithm does a much better job at identifying the main flame front and product pockets (shown in red color) than the standard Otsu segmentation algorithm in Fig. 5 (s1-6). However, a number of holes appear in the burnt gas region as a result of slight noise amplification in low SNR regions, which overestimates the number of reactant pockets. Hence, a pocket identification stage is implemented to distinguish real reactant pockets from noise. First, all isolated pockets in the burnt gas region (shown in gray in Fig. 7a) are highlighted and labeled as “potential” flame pockets. Second, two-dimensional gradients magnitudes are computed across all potential flame pockets. Third, the mean gradient magnitude across each pocket is computed and a $20\%$ gradient threshold is applied. This way, all pockets whose mean gradient magnitude is below the threshold are suppressed which ensures only real pockets are preserved (Fig. 7c). This approach was found to produce excellent results as erroneous pockets are typically present in uniform low-SNR regions characterized by low gradient magnitudes $|\nabla P_{rp}|$. Finally, the true reactant pockets are combined with the main front and product pockets to yield the final flame front. By comparing the obtained flame fronts *F* in Fig. 7d to those obtained by Otsu segmentation in Fig. 5 (s1-6), one can clearly notice the perks of the proposed contrast enhancement scheme. The flame fronts obtained are qualitatively similar to those computed by Canny edge detection in Fig. 5 (e1-6) which is an additional testament to the fidelity of the proposed pre-processing scheme.

### A first quantification of segmentation accuracy

We mentioned previously that the accuracy of segmentation can be improved by adaptive contrast enhancement and illustrated that for images containing a number of multi-scale pockets. In this section, we go one step further and quantify the merit of this pre-processing scheme. The objective is twofold: first, to optimize Otsu contours and ensure they are as close as possible to the location of peak gradients highlighted by the Augmented Canny algorithm, then second, quantify their localization error with increasing hydrogen enrichment by means of Euclidean distances. This is illustrated in Fig. 8 where probability histograms of normalized Euclidean distances $d/\delta _L$ are shown for a variable hydrogen enrichment with and without CLAHE. A perfect histogram would be a delta function at zero.

**Fig. 9:**
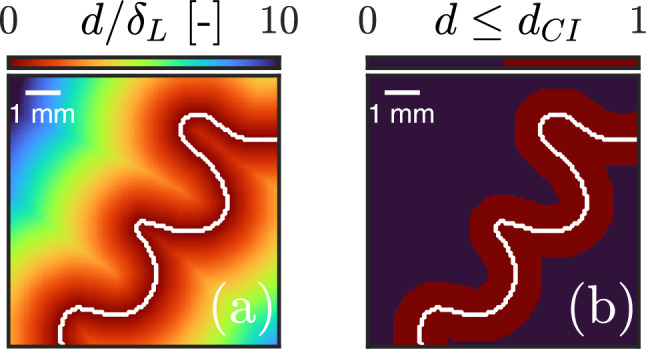
Visual interpretation of the estimated CI for a flame front from the $70\%$ $H_2$ condition. **a** Euclidean distance *d* map of a flame front obtained by Enhanced Otsu segmentation (traced in white) normalized by the laminar flame thickness $\delta _L$, **b** Binary confidence interval (colored in red) obtained by thresholding the Euclidean distance map by the upper limit of the $90\%$ CI $d_{\textrm{CI}}/\delta _L = 1.5$

A first observation can be made on the effect of hydrogen enrichment on the shape of the histograms, which appear to flatten with increasing hydrogen content due to a higher variance. This pushes the upper limit of the $90\%$ confidence interval (CI), $d_{\textrm{CI}}$, beyond unity due to the intense wrinkling induced by hydrogen and the prevalence of thin elongated flame fingers hard to capture by the algorithm. Overall, there is strong evidence that the proposed pre-processing scheme improves the accuracy of segmentation. Notice, for example, how the $d_{\textrm{CI}}$ limit is decreased by a factor of three in the methane-air case, resulting in $90\%$ of points being within a distance of half the laminar flame thickness from peak OH gradients. The same can be said for the hydrogen-enriched methane-air flames where factors of 15 and 12 were recorded for $40\%$ and $70\%$, respectively. Indeed, while the upper limit plateaus at $d_{\textrm{CI}} = 1.5 \delta _L$ when the proposed scheme is used, it can reach exceedingly large values ($d_{\textrm{CI}} > 10 \delta _L$) when CLAHE is omitted in the hydrogen-enriched cases. A visual interpretation of this confidence interval is presented in Fig. 9 for a flame front from the $70\%$ hydrogen case. The flame front is estimated using Enhanced Otsu segmentation, and its Euclidean distance transform is then computed. By thresholding the Euclidean distance map with the measured $90\%$ CI (i.e: $d_{\textrm{CI}} = 1.5\delta _L$ for the hydrogen-enriched cases), one can draw a binary region around the Otsu flame front where the peak OH gradients are likely to reside. This concept is the foundation of the hybrid approach presented in the next section.

### Filtered Canny algorithm

**Fig. 10:**
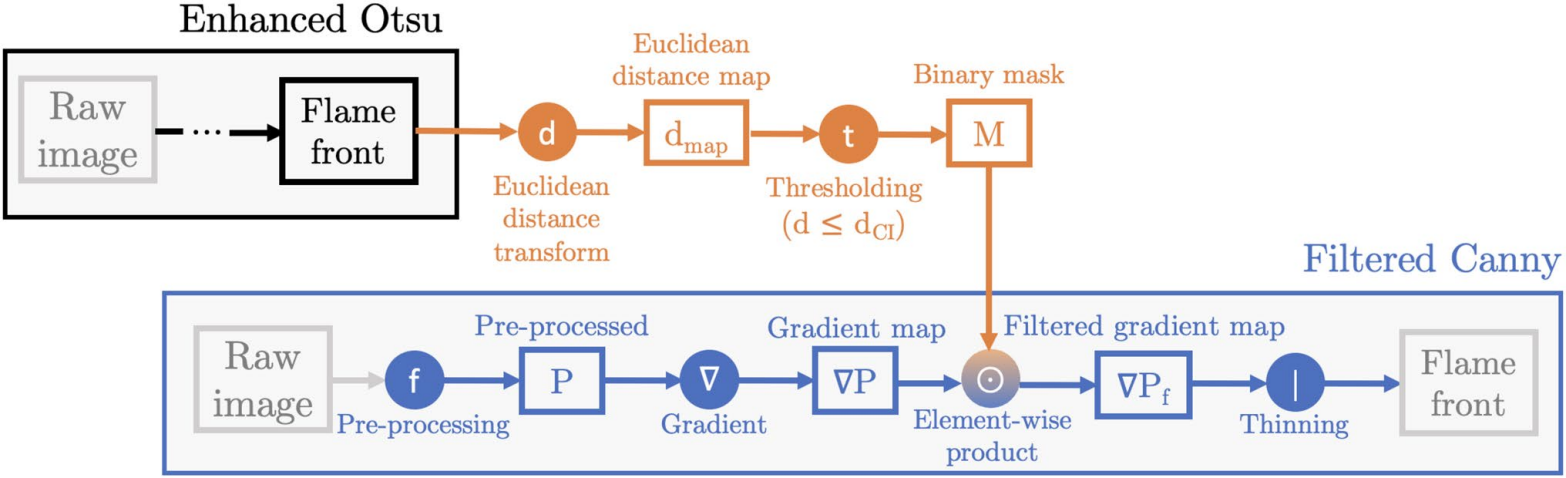
Flowchart of the Filtered Canny algorithm

**Fig. 11:**
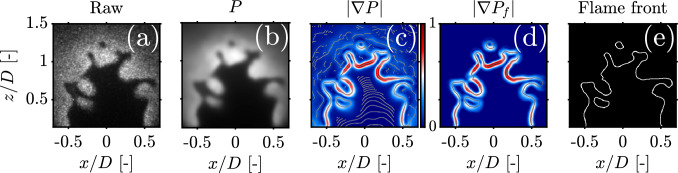
Illustration of the Filtered Canny edge detection process: **a** Raw OH-PLIF image, **b** Pre-processed image using the bilateral-NLD filtering scheme, **c** Gradient map of the pre-processed image $|\nabla P|$, its thinned version is overlayed in gray, **d** Gradient map filtered using the binary mask $|\nabla P_f|$, its thinned version is overlayed in gray, **e** Final binary flame front obtained by thinning the filtered gradient map $|\nabla P_f|$

The Filtered Canny algorithm revolves around using the approximate position of the flame front obtained by Enhanced Otsu segmentation to filter the image’s gradient map prior to thinning. As can be seen in Fig. 10, this suppresses the need for hysteresis thresholding.

A preliminary flame front is first obtained using the Enhanced Otsu algorithm, then used to construct a binary $90\%$ CI in the same fashion as in Fig. 9. The upper limits of the confidence interval $d_{\textrm{CI}}$ are determined from the results in Fig. 8 for each experimental condition: $d_{\textrm{CI}} = 0.5\delta _L$ for the methane-air case, and $d_{\textrm{CI}} = 1.5\delta _L$ for both hydrogen-enriched cases. One obtains a binary mask which will be used to filter the gradient map later on. In parallel, the Filtered Canny algorithm follows the same initial steps as the original algorithm in Fig. 1. A pre-processing scheme combining two edge-preserving filters, the bilateral and NLD filters, is used to filter the image. The bilateral filter’s standard deviation and degree of smoothing are set to 10 and 500, respectively, to ensure a good balance between smoothing quality and computation time, and a moderate value of $N = 50$ is used for NLD. The two-dimensional gradient map is computed from the pre-processed image and then filtered using the binary mask *M* obtained from segmentation. Mathematically, this is treated as a simple element-wise (Hadamard) matrix product, denoted using the circled dot symbol “$\odot$,” and yields a filtered gradient map $|\nabla P_f|$:

7 $$\begin{aligned} |\nabla P_f| = M\odot |\nabla P| \end{aligned}$$

In doing so, only gradients residing in the super - equilibrium region are kept and later thinned to produce the final flame front. Conversely, gradients in the reactant and product sides are suppressed completely, which eliminates the need for hysteresis thresholding. Flame edges are hence extracted from the filtered gradient map regardless of their gradient magnitudes to produce a thin and continuous flame front. This is illustrated in Fig. 11 for a PLIF snapshot from the $70\%$ hydrogen enrichment case. Notice how the thinned flame edge obtained from the filtered gradient map in Fig. 11d captures both the flame front and isolated flame pockets and is unpolluted by noisy spurs which are prevalent in the unfiltered gradient map shown in Fig. 11c. By using the CI as a spatial filter, one is able to detect the flame front without resorting to the problematic hysteresis thresholding stage. In a handful images, small discontinuities ($<5\,$px) can, however, be encountered across the flame edge which are inherent to the Canny algorithm. These gaps were filled using a simple directional edge linker similar to the one used in Sweeney and Hochgreb (2009), with more information in “Appendix.” All in all, the Filtered Canny method is identical to the original Canny algorithm with hysteresis thresholding omitted and replaced by a masking stage before thinning. Since no hysteresis thresholds are required, the proposed algorithm is fully unsupervised and does not require any user intervention. The only parameter required to obtain the final flame front is in fact the upper limit $d_{\textrm{CI}}$ for which suitable ranges are documented. In the next section, we evaluate the accuracy and computation time of the Filtered Canny algorithm against its high-performance Augmented counterpart.

## Performance evaluation

**Table 3:** Comparison of ROC statistics for a variable hydrogen enrichment

Method	$0\%$ $H_2$		$40\%$ $H_2$		$70\%$ $H_2$	
	Precision	Recall	Precision	Recall	Precision	Recall
Filtered Canny	$0.99^{1.00}_{0.96}$	${0.99}^{1.00}_{0.95}$	${0.95}^{1.00}_{0.84}$	${0.96}^{1.00}_{0.88}$	${0.95}^{1.00}_{0.83}$	${0.93}^{0.99}_{0.87}$
Enhanced Otsu	$0.22^{0.27}_{0.17}$	${0.23}^{0.28}_{0.18}$	${0.20}^{0.26}_{0.14}$	${0.21}^{0.27}_{0.15}$	${0.17}^{0.22}_{0.12}$	${0.18}^{0.23}_{0.13}$
Otsu (standard)	$0.12^{0.18}_{0.06}$	${0.12}^{0.18}_{0.06}$	${0.10}^{0.17}_{0.03}$	${0.10}^{0.16}_{0.04}$	${0.14}^{0.21}_{0.07}$	${0.15}^{0.21}_{0.09}$

68.3% confidence intervals for precision and recall are shown in the format: ${\mu }^{\mu +\sigma }_{\mu -\sigma }$, where $\mu$ and $\sigma$ represent the mean and standard deviation, respectively

The performance of the Filtered Canny algorithm is evaluated against the ground-truth Augmented Canny algorithm based on accuracy (localization errors and curvature) and computation time using the metrics introduced in Sect. 3.5.

### Localization errors

Localization errors are evaluated using precision and recall. Both quantities are measured on an image-by-image basis, so reliable statistics can be computed. The results are provided in Table 3 for the Filtered Canny algorithm at variable hydrogen content. Results for Enhanced and standard Otsu segmentation are also provided for reference. As expected, the latter performs the poorest yielding low values of precision and recall for all three experimental conditions. When CLAHE is used, both precision and recall increase by a factor of 2 for $0\%$ and $40\%$ hydrogen enrichment and barely increase in the $70\%$ case. Both metrics remain relatively low with only one in four pixels, at best, perfectly overlapping with its counterpart in the ground-truth. In light of the statistics presented previously in Sect. 4.2, we conclude that although CLAHE reduces the mean shift of Otsu contours relative to the position of peak OH gradients, the overlap remains low and unsatisfactory, especially with hydrogen addition. The Filtered Canny algorithm, on the other hand, performs remarkably well and yields high values of precision and recall. Mean values remain in the $90\%$ range when hydrogen is added with a slight increase in standard deviation. These results showcase the algorithm’s ability to successfully detect flame fronts with limited over and underestimation of flame edge points.

### Curvature

**Fig. 12:**
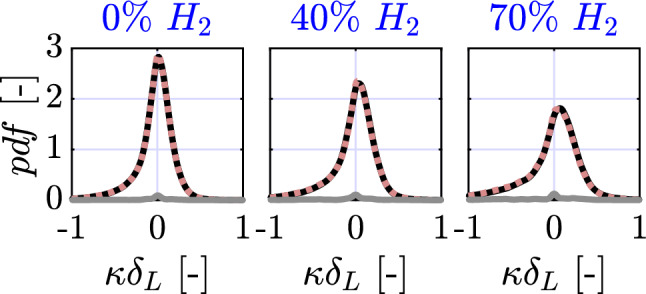
Probability density function of two-dimensional curvature $\kappa$ normalized by the laminar flame thickness $\delta _L$ for the Augmented (solid dark lines) and Filtered (dashed red lines) Canny algorithms for a variable hydrogen enrichment. The absolute difference between pdfs is shown in solid gray color

**Table 4:** Comparison of algorithm runtimes for a variable hydrogen enrichment

Method	$0\%$ $H_2$		$40\%$ $H_2$		$70\%$ $H_2$	
	*t* (s)	$t_{\textrm{total}}$ (h)	*t* (s)	$t_{\textrm{total}}$ (h)	*t* (s)	$t_{\textrm{total}}$ (h)
Augmented Canny	$10.5^{11.9}_{9.2}$	56	${12.2}^{13.6}_{10.7}$	65	${10.8}^{12.5}_{9.1}$	58
Filtered Canny	$0.35^{0.38}_{0.32}$	2	${0.31}^{0.32}_{0.30}$	2	${0.34}^{0.36}_{0.32}$	2

68.3% confidence intervals for computation time per image *t* are shown in the format: ${\mu }^{\mu +\sigma }_{\mu -\sigma }$, where $\mu$ and $\sigma$ represent the mean and standard deviation, respectively. The expected total computation time $t_{\textrm{total}}$ required to reach fully converged flame statistics is computed from the mean value of *t* and rounded to the nearest hour for better visibility

Probability density functions (pdf) of two-dimensional curvature are computed for both the Filtered and Augmented Canny algorithms and are presented in Fig. 12. As can be seen, the obtained curvature pdfs are identical which showcases the Filtered Canny algorithm’s ability to capture the geometry of the flame front with a great degree of accuracy. As hydrogen is added to the mixture, the flame experiences a higher degree of wrinkling which results in a broader range of curvatures across the flame front. As can be observed in Fig. 12, the proposed algorithm is able to capture this transition in the global geometry of the flame front and remains just as accurate at maximum hydrogen enrichment. In all three conditions, the absolute difference is symmetric and highest at the point of zero curvature due to a slight shift between Augmented and Filtered pdf modes. As the study was only limited to 500 flame fronts per condition due to the time required to compute ground-truths, we expect these small differences to disappear if a larger sample size of images is used. Overall, the results suggest that the flame fronts obtained using the Filtered Canny algorithm are of comparable accuracy to its Augmented counterpart.

We conclude that accurate mapping of turbulent flame fronts can be achieved using the proposed hybrid scheme. The algorithm is able to detect flame edge points with comparable accuracy to its sophisticated high-performance alternative and identifies the global shape of complex and highly convoluted flame fronts faithfully.

### Computation time

Finally, the execution times of both the Filtered and Augmented Canny algorithms are recorded for each experimental condition on an image-by-image basis. Both algorithms are implemented in MATLAB^®^ (R2021b) which is commonly used for post-processing tasks in experimental combustion. The same implementation and settings of the Augmented algorithm presented in Sweeney and Hochgreb (2009) are used for consistency, with $\sigma$ kept constant ($\sigma = 2$) and both $t_{\textrm{low}}$ and $t_{\textrm{high}}$ varied in the 0.1 to 0.9 range with a small step size of $\varDelta t = 0.1$. The Filtered algorithm does not require input parameters, and mask construction is facilitated by MATLAB^®^’s built-in function bwdist for time-efficient computation of 2D Euclidean distance transforms. Computation times are reported in Table 4 and were all recorded on the same 8-core CPU Macbook Air M1 workbench. The results highlight the impressive computational speed of the proposed algorithm which is, on average, at least 30 times quicker than its Augmented counterpart. The Filtered algorithm can process up to three instantaneous OH-PLIF snapshots per second, while the Augmented algorithm needs at least ten seconds to process a single snapshot. Although the analysis was restricted to 500 flame fronts per condition in this study, a larger sample size of images is typically required to compute fully converged flame statistics relevant to the study of turbulent flames. In fact, a residual analysis showed that around 19,300 instantaneous flame fronts were required to reach full convergence of flame statistics. The expected total computation time $t_{\textrm{total}}$ of each algorithm was therefore estimated using mean runtimes *t*. Using the Augmented algorithm, one should expect total runtimes between 56 and 65 h, while the Filtered algorithm is expected to process a full data set (19,300 images) in record time, estimated at 2 h approximately. The reported runtimes include the edge detection stage only. Therefore, any further processing (i.e., instantaneous curvatures, flame surface density, etc.) will most likely increase the total computation time. Thus, it is evident the proposed hybrid algorithm, in addition to being just as accurate, is quicker and more time-efficient than the high-performance Augmented algorithm. This facilitates the processing of larger data sets of turbulent and highly wrinkled flame fronts within reasonable computation times.

## Conclusion

The paper presents a hybrid and unsupervised approach to flame front detection in noisy PLIF images combining Otsu segmentation and Canny edge detection: the Filtered Canny algorithm. An adaptive contrast enhancement method, CLAHE, is proposed to improve the accuracy of segmentation with little increase in computational overhead. The contours obtained using segmentation can then be used to filter two-dimensional gradient maps in the Canny edge detection algorithm, which suppresses the need for hysteresis thresholding and hence supervision. The proposed hybrid Filtered Canny algorithm is evaluated against a high-performance alternative, the Augmented Canny algorithm (Sweeney and Hochgreb 2009), and is found to produce results of comparable accuracy with a significant reduction in computational time (factor of 30). The method produces excellent results in finely wrinkled hydrogen-rich flames and is able to pick out all crucial geometric features and isolated flame pockets. Additionally, the proposed algorithm can be easily implemented in MATLAB software using standard libraries which makes it accessible. The result of this work highlights the performance of simpler, computationally inexpensive flame front detection methods, provided that compatible pre-processing techniques are applied. Most importantly, this study demonstrates that segmentation and edge detection, which are both typically looked at as rivals in flame front detection tasks, can in fact benefit from each other to produce more accurate results at low computational cost.

### Supplementary Information

Below is the link to the electronic supplementary material.

Supplementary file 1 (mp4 12053 KB)

## Figures and Tables

**Fig. 13:**
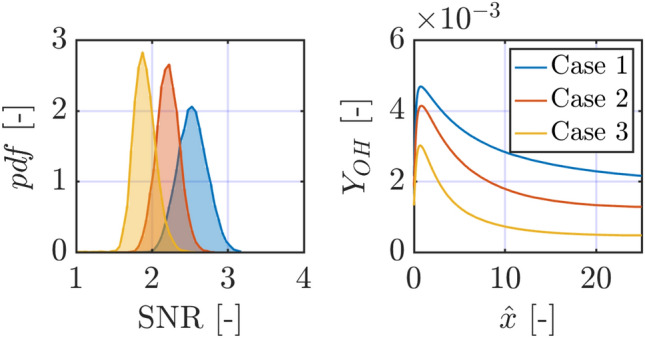
(Left): Probability density functions of signal-to-noise ratios obtained from a sample size of 10,000 raw OH-PLIF snapshots per experimental condition. (Right): OH mass fraction $Y_{OH}$ as a function of the normalized distance from the flame front ${\hat{x} = ({x - x_{max(|\nabla Y_{OH}|)}})/{\delta _L}}$ for each experimental condition, obtained from 1D laminar flame simulations. The normalized distance is defined here such that ${\hat{x} = 0}$ corresponds to the flame front location (peak OH gradients), with positive values ${\hat{x}>0}$ being the product region downstream the flame front

**Fig. 14:**
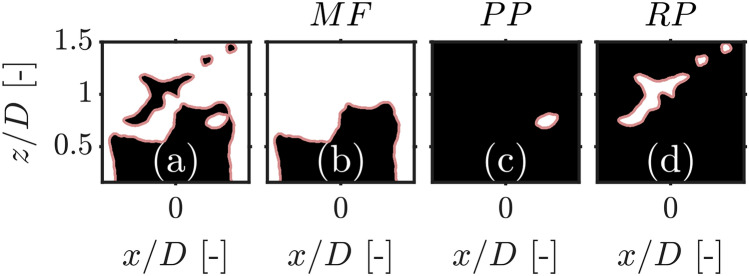
Illustration of a flame object classification operation applied to a binarized image obtained by segmentation: **a** Initial binary image with all flame objects highlighted in red color, **b** Main flame front (MF), **c** Product pocket (PP), **d** Reactant pockets (RP)

**Fig. 15:**
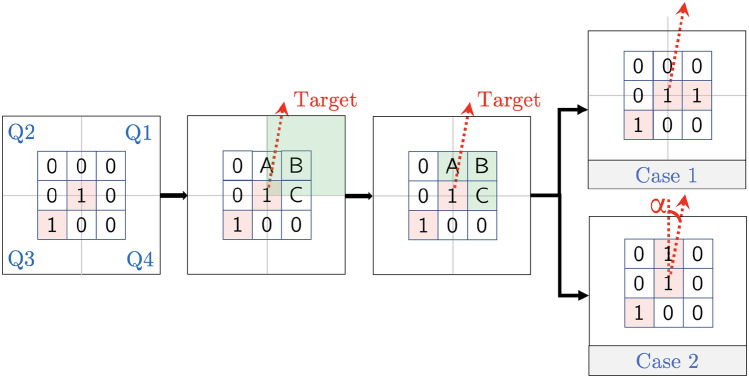
Illustration of the edge linking process

## Data Availability

An open-source sample code is provided in a separate GitLab repository: https://gitlab.developers.cam.ac.uk/eng/div-a-public/filtered-canny/.

## Appendix

### Signal-to-noise ratio computation

To compute the SNR, a total of 10,000 images per condition were binarized into products and reactants after edge detection. The SNR was then computed for each instantaneous image using the expression (Sweeney and Hochgreb 2009):

8 $$\begin{aligned} SNR = \frac{\mu _{products} - \mu _{reactants}}{\sigma _{products}} \end{aligned}$$

where $\mu$ and $\sigma$ correspond to the mean and standard deviation of OH intensities in the product (burnt) or reactant (unburnt) regions. Probability density functions of the signal-to-noise ratio are provided in Fig. 13 for each experimental condition and show a shift toward lower SNR values with increasing hydrogen addition. This was found to be due to decreasing values of mean OH intensities in the post-combustion region $\mu _{products}$ when moving from case 1 to 3. This is consistent with the results of the 1D laminar flame simulations provided in Fig. 13 where the mass fraction of the OH radical in the product region downstream the flame front decreases from case 1 to 3. The shift observed in SNR pdfs is also due to a slight increase in standard deviations $\sigma _{products}$ from case 1 to 3. This is likely due to the thermodiffusive effects mentioned previously in Sect. 3.3 which lead to higher variations in OH intensities in the product region directly downstream the flame front. This is particularly pronounced in case 3 due to high hydrogen enrichment and a low equivalence ratio which decreases the effective Lewis number of the mixture (Bouvet et al. 2013).

### Flame object classification

In the Enhanced Otsu segmentation algorithm, a pocket identification stage is implemented to discard erroneous pockets caused by slight noise amplification in the burnt gas region. Prior to this stage, flame objects are classified into one of three categories: main front, product pockets, and reactant pockets (Fig. 14). This step is implemented using MATLAB^®^’s built-in capabilities (via the Image Processing Toolbox) and the steps are as follows:

1. Discarding pockets: Starting from the initial binary image obtained via segmentation, all the gaps in the burnt gas region are filled using the function imfill.
2. Main front identification: The binary image obtained after discarding the pockets is then considered. The function bwconncomp is used to identify all connected objects in the image. Each object represents a collection of 8 - connected non-zero pixels in the image which make up one isolated area. The largest object is typically the burnt gas area, while other objects correspond to product pockets. The main flame front is thus obtained by selecting the perimeter of the largest object (highest pixel count) in the image using the function bwperim.
3. Product pocket identification: The remaining objects identified in the previous steps are then highlighted individually. Their perimeters are traced in a similar fashion and they are then classified as product pockets.
4. Reactant pocket identification: Finally, reactant pockets are obtained by subtracting the main front and product pocket from the perimeter of the initial binary image (i.e., before step 1 was applied).

### Flame edge linking

Small discontinuities of the order of a few pixels can persist in binary flame edges (1’s and 0’s) after flame front detection using the Canny edge detection algorithm. If continuous edges are needed (i.e., to binarize the image into reactants and products), a separate edge linking strategy is needed. An approach similar to the one described in Sweeney and Hochgreb (2009) is followed in this work with a number of modifications. The steps of the edge linking script are as follows:

1. Locating discontinuities: The script starts by identifying all endpoints corresponding to discontinuities across the flame front. This is done by locating flame front pixels with no more than one non-zero adjacent pixel in the 8-neighborhood.
2. Finding pairs: Endpoints are paired up based on their Euclidean distances. This comes down to an optimization problem: we try to find the optimal and unique pairs of points such that the Euclidean distance between pairs is minimized.
3. Edge tracing: The algorithm proceeds to link each pair of endpoints in an iterative and pixelwise manner. At each iteration, the direction of the target endpoint is used to select a quadrant in the 8 - neighborhood of the current pixel (Fig. 15). The three adjacent pixels within the same quadrant (pixels A, B, and C in Fig. 15) are then highlighted and their gradient magnitudes are compared. Typically, one of two cases will arise:
  - Case 1: A unique maximum is identified (i.e., the gradient magnitude of point C in Fig. 15 is highest). The pixel with the highest gradient magnitude is classified as a flame edge pixel and thus assigned a value of 1.
  - Case 2: There is a non-unique maximum (i.e., the gradient magnitudes of points A, B, and C in Fig. 15 are equal). In this case, the pixel which minimizes the search angle $\alpha$ relative to the direction of the target (i.e., pixel A in Fig. 15) is chosen instead and then assigned a value of 1.
  The location is then shifted to the newly identified flame edge pixel and the process is repeated until the target pixel is reached.

## References

[CR1] Azam ASB, Malek AA, Ramlee AS, Suhaimi NDSM, Mohamed N (2020) Segmentation of breast microcalcification using hybrid method of Canny algorithm with Otsu thresholding and 2D Wavelet transform. In: 2020 10th IEEE international conference on control system, computing and engineering (ICCSCE), pp 91–96. 10.1109/ICCSCE50387.2020.9204950

[CR2] Barlow RS (2007). Laser diagnostics and their interplay with computations to understand turbulent combustion. Proc Combust Inst.

[CR3] Bayley AE, Hardalupas Y, Taylor AMKP (2012). Local curvature measurements of a lean, partially premixed swirl-stabilised flame. Exp Fluids.

[CR4] Bell JB, Cheng RK, Day MS, Shepherd IG (2007). Numerical simulation of Lewis number effects on lean premixed turbulent flames. Proc Combust Inst.

[CR5] Berger L, Kleinheinz K, Attili A, Pitsch H (2019). Characteristic patterns of thermodiffusively unstable premixed lean hydrogen flames. Proc Combust Inst.

[CR6] Berger L, Grinberg M, Jürgens B, Lapenna PE, Creta F, Attili A, Pitsch H (2022). Flame fingers and interactions of hydrodynamic and thermodiffusive instabilities in laminar lean hydrogen flames. Proc Combust Inst.

[CR7] Bouvet N, Halter F, Chauveau C, Yoon Y (2013). On the effective Lewis number formulations for lean hydrogen/hydrocarbon/air mixtures. Int J Hydrogen Energy.

[CR8] Boxx I, Slabaugh C, Kutne P, Lucht R, Meier W (2015). 3 khz piv/oh-plif measurements in a gas turbine combustor at elevated pressure. Proc Combust Inst.

[CR9] Burger W, Burge MJ (2016). Digital image processing: an algorithmic introduction using java.

[CR10] Canny J (1986). A computational approach to edge detection. IEEE Trans Pattern Anal Mach Intell.

[CR11] Chrystie RSM, Burns IS, Hult J, Kaminski CF (2008). On the improvement of two-dimensional curvature computation and its application to turbulent premixed flame correlations. Meas Sci Technol.

[CR12] Coppola G, Gomez A (2009). Experimental investigation on a turbulence generation system with high-blockage plates. Exp Therm Fluid Sci.

[CR13] Day M, Bell J, Bremer P-T, Pascucci V, Beckner V, Lijewski M (2009). Turbulence effects on cellular burning structures in lean premixed hydrogen flames. Combust Flame.

[CR14] Fan Q, Liu X, Xu L, Subash AA, Brackmann C, Aldén M, Bai X-S, Li Z (2022). Flame structure and burning velocity of ammonia/air turbulent premixed flames at high Karlovitz number conditions. Combust Flame.

[CR15] Goodwin DG, Moffat HK, Schoegl I, Speth RL, Weber BW (2022) Cantera: an object-oriented software toolkit for chemical kinetics, thermodynamics, and transport processes. Version 2.6.0. 10.5281/zenodo.6387882

[CR16] Gulder OL, Smallwood GJ (2007). Flame surface densities in premixed combustion at medium to high turbulence intensities. Combust Sci Technol.

[CR17] Halter F, Chauveau C, Gökalp I, Veynante D (2009). Analysis of flame surface density measurements in turbulent premixed combustion. Combust Flame.

[CR18] Haq M, Sheppard C, Woolley R, Greenhalgh D, Lockett R (2002). Wrinkling and curvature of laminar and turbulent premixed flames. Combust Flame.

[CR19] Hartung G, Hult J, Balachandran R, Mackley MR, Kaminski CF (2009). Flame front tracking in turbulent lean premixed flames using stereo PIV and time-sequenced planar LIF of OH. Appl Phys B.

[CR20] Howarth T, Aspden A (2022). An empirical characteristic scaling model for freely-propagating lean premixed hydrogen flames. Combust Flame.

[CR21] Kobayashi H, Seyama K, Hagiwara H, Ogami Y (2005). Burning velocity correlation of methane/air turbulent premixed flames at high pressure and high temperature. Proc Combust Inst.

[CR22] Kraemer H (1992). Evaluating medical tests: objective and quantitative guidelines.

[CR23] Kushwaha A, Kasthuri P, Pawar SA, Sujith RI, Chterev I, Boxx I (2021). Dynamical characterization of thermoacoustic oscillations in a hydrogen-enriched partially premixed swirl-stabilized methane/air combustor. J Eng Gas Turb Power.

[CR24] Lawn C, Schefer R (2006). Scaling of premixed turbulent flames in the corrugated regime. Combust Flame.

[CR25] Malbois P, Salaün E, Vandel A, Godard G, Cabot G, Renou B, Boukhalfa A, Grisch F (2019). Experimental investigation of aerodynamics and structure of a swirl-stabilized kerosene spray flame with laser diagnostics. Combust Flame.

[CR26] Malm H, Sparr G, Hult J, Kaminski CF (2000). Nonlinear diffusion filtering of images obtained by planar laser-induced fluorescence spectroscopy. J Opt Soc Am A.

[CR27] Manosh Kumar R, Chterev I, Stepien D, Sirignano M, Emerson BL, Yi T, Jiang N, Fugger CA, Hsu PS, Felver J, Roy S, Gord JR, Lieuwen TC (2019) Characterization of transient blowout dynamics of a swirl stabilized flame using simultaneous OH and CH$$_{\rm 2}$$O PLIF. In: AIAA Scitech 2019 Forum. American Institute of Aeronautics and Astronautics, San Diego. 10.2514/6.2019-2245

[CR28] McManus TA, Sutton JA (2020). Simultaneous 2D filtered Rayleigh scattering thermometry and stereoscopic particle image velocimetry measurements in turbulent non-premixed flames. Exp Fluids.

[CR29] Mohammadnejad S, An Q, Vena P, Yun S, Kheirkhah S (2021). Contributions of flame thickening and extinctions to a heat release rate marker of intensely turbulent premixed hydrogen-enriched methane-air flames. Combust Flame.

[CR30] Otsu N (1979). A threshold selection method from gray-level histograms. IEEE Trans Syst Man Cybern.

[CR31] Pareja J, Lipkowicz T, Inanc E, Carter CD, Kempf A, Boxx I (2022). An experimental/numerical investigation of non-reacting turbulent flow in a piloted premixed Bunsen burner. Exp Fluids.

[CR32] Perona P, Malik J (1990). Scale-space and edge detection using anisotropic diffusion. IEEE Trans Pattern Anal Mach Intell.

[CR33] Pfadler S, Beyrau F, Leipertz A (2007). Flame front detection and characterization using conditioned particle image velocimetry (CPIV). Opt Express.

[CR34] Pizer SM, Amburn EP, Austin JD, Cromartie R, Geselowitz A, Greer T, Zuiderveld A (1986) Adaptive histogram equalization and its variations p 14

[CR35] Qi Y, Wang Y, Li Y, Wang J, He X, Wang Z (2019). Auto-ignition characteristics of end-gas in a rapid compression machine under super-knock conditions. Combust Flame.

[CR36] Reisenhofer R, Kiefer J, King EJ (2016). Shearlet-based detection of flame fronts. Exp Fluids.

[CR37] Setiawan BD, Rusydi AN, Pradityo K (2017) Lake edge detection using Canny algorithm and Otsu thresholding. In: 2017 International symposium on geoinformatics (ISyG), pp 72–76. 10.1109/ISYG.2017.8280676

[CR38] Skiba AW, Carter CD, Hammack SD, Driscoll JF (2022). Premixed flames subjected to extreme levels of turbulence part II: surface characteristics and scalar dissipation rates. Combust Flame.

[CR39] Smith GP, Golden DM, Frenklach M, Moriarty NW, Eiteneer B, Goldenberg M, Bowman CT, Hanson RK, Song S, Gardiner WC, Jr, Lissianski VV, Qin Z (1999) Gri-mech 3.0. http://www.me.berkeley.edu/gri_mech/

[CR40] Stöhr M, Boxx I, Carter CD, Meier W (2012). Experimental study of vortex–flame interaction in a gas turbine model combustor. Combust Flame.

[CR41] Sweeney M, Hochgreb S (2009). Autonomous extraction of optimal flame fronts in oh planar laser-induced fluorescence images. Appl Opt.

[CR42] Sweeney M, Hochgreb S, Dunn M, Barlow R (2011). A comparative analysis of flame surface density metrics inpremixed and stratified flames. Proc Combust Inst.

[CR43] Tachibana S, Zimmer L, Suzuki K (2004) Flame front detection and dynamics using PIV in a turbulent premixed flame. In: 12th international symposium on applications of laser techniques to fluid mechanics, p 12. http://ltces.dem.ist.utl.pt/lxlaser/lxlaser2004/pdf/paper_04_4.pdf

[CR44] Tyagi A, Boxx I, Peluso S, O’Connor J (2019). Statistics and topology of local flame–flame interactions in turbulent flames. Combust Flame.

[CR45] Tyagi A, Boxx I, Peluso S, O’Connor J (2020). Pocket formation and behavior in turbulent premixed flames. Combust Flame.

[CR46] Verbeek A, Jansen W, Stoffels G, van der Meer T (2013) Improved flame front curvature measurements for noisy oh-lif images. In: 8th World conference on experimental heat transfer, fluid mechanics, and thermodynamics, ExHFT-8 Lisbon, Portugal. https://ris.utwente.nl/ws/portalfiles/portal/15075681/ExHFT-8_Verbeek.pdf

[CR47] Wabel TM, Skiba AW, Driscoll JF (2017). Turbulent burning velocity measurements: extended to extreme levels of turbulence. Proc Combust Inst.

[CR48] Wabel TM, Skiba AW, Temme JE, Driscoll JF (2017). Measurements to determine the regimes of premixed flames in extreme turbulence. Proc Combust Inst.

[CR49] Zhang M, Wang J, Xie Y, Wei Z, Jin W, Huang Z, Kobayashi H (2014). Measurement on instantaneous flame front structure of turbulent premixed CH4/H2/air flames. Exp Therm Fluid Sci.

[CR50] Zheng Y, Weller L, Hochgreb S (2022). Instantaneous flame front identification by Mie scattering vs OH PLIF in low turbulence Bunsen flame. Exp Fluids.

[CR51] Zuiderveld K (1994). Contrast limited adaptive histogram equalization.

